# A Voice Sign in Chorea

**Published:** 1914-07

**Authors:** 


**A Voice Sign in Chorea.** Swift in the American Journal of Diseases of Children, June, 1914, claims priority for a new diagnostic sign in chorea consisting in a pretty constant voice change of rise in pitch and increase in intensity accompanying choreic movements. This is demonstrated graphically by the kymograph which records changes in the air pressure and vocal cord vibrations. He proves from the literature that this is an entirely new method and the first one in which a recording appartus was used.—C. G. L-W.

